# On the Quantity of Carbonic Acid Exhaled from the Lungs in 24 Hours

**Published:** 1845-11

**Authors:** 


*On the Quantity of Carbonic Acid exhaled from the Lungs in Twenty-Four Hours.*—The results of M. Scharling's experiments on this subject are the following:—

1. The quantity of carbonic acid exhaled from the lungs varies considerably in different periods of the day.
2. These variations are attributable, in part, to the varying power or faculty of the respiratory organs to change the inspired air into the gaseous product; and in part also to the unequal movement of the circulation—the state of which is very sensibly affected by that of the process of digestion.
3. *Cæteris paribus*, a person exhales a greater quantity of carbonic acid when full than when he is fasting, and also more during waking than sleeping.
4. Men exhale more than women of the same age, and children more than adults.
5. In various states of sickness, there is considerably less exhaled than during health.—*Schmidt's Jahrbucher.*

